# Insights into Assessing the Genetics of Endometriosis

**DOI:** 10.1007/s13669-012-0016-5

**Published:** 2012-06-15

**Authors:** Nilufer Rahmioglu, Stacey A. Missmer, Grant W. Montgomery, Krina T. Zondervan

**Affiliations:** 1 Wellcome Trust Centre for Human Genetics, University of Oxford, Oxford, UK; 2 Department of Obstetrics, Gynecology, and Reproductive Biology, Brigham and Women’s Hospital and Harvard Medical School, Boston, MA USA; 3 Channing Laboratory, Department of Medicine, Brigham and Women’s Hospital and Harvard Medical School, Boston, MA USA; 4 Department of Epidemiology, Harvard School of Public Health, Boston, MA USA; 5 Molecular Epidemiology, Queensland Institute for Medical Research, Brisbane, Australia; 6 Nuffield Department of Obstetrics and Gynaecology, University of Oxford, Oxford, UK

**Keywords:** Endometriosis, Genetics, Heritability, Genetic epidemiology, Candidate gene, Genome-wide association study, Linkage study

## Abstract

Endometriosis is a complex disease arising from the interplay between multiple genetic and environmental factors. The genetic variants potentially underlying the hereditary component of endometriosis have been widely investigated through hypothesis-driven candidate gene studies, an approach that generally has proven to be inherently difficult and problematic for a number of reasons. Recently, through major collaborative efforts in the endometriosis research field, hypothesis-free genome-wide approaches have started to provide new insights into potential pathways leading to development of endometriosis, as well as highlighting the phenotypic heterogeneity of the condition. This review summarizes the most recent studies investigating the genetic variation contributing to endometriosis, with a particular focus on genome-wide approaches, and discusses promising future directions of genetic research.

## Introduction

Endometriosis is a common estrogen (E)-dependent gynecological disorder, characterized by presence of tissue resembling endometrium tissue outside the uterine cavity. Typical locations for the ectopic deposits are on the pelvic peritoneum, the ovaries, uterosacral ligaments, pouch of Douglas, and the rectovaginal septum. Endometriosis has been associated with chronic pelvic pain, reduced fertility, and severe dysmenorrhea [1, 2]. Definitive diagnosis can only be established through surgery, and, though other classifications exist, the disease is most commonly staged using the revised American Fertility Society (rAFS) classification, based on the total surface size of the lesions, presence of adhesions, and ovarian lesions [3]. Because endometriosis is E-dependent, it occurs almost exclusively in women of reproductive age [4]. Because of the need for surgical diagnosis, the exact population prevalence is unknown. Based on community prevalence rates of pelvic pain and infertility, the prevalence of endometriosis is estimated to be about 5–10 % in premenopausal women [5], increasing to 35–50 % in women undergoing laparoscopy for pelvic pain and infertility [1, 6].

The etiology of endometriosis remains unclear. The most widely accepted mechanism for development of the peritoneal endometriotic lesions is via retrograde menstruation [7]. However, menstrual debris is present in the peritoneal cavity of up to 90 % of the menstruating women [8]. Possible explanations for adhesion and growth of endometriotic lesions in only some women include increased exposure to menstrual debris (e.g., increased menstrual flow, shorter cycle length), abnormal eutopic endometrium, altered peritoneal environment, reduced immune surveillance, and increased angiogenic capacity [9–11]. It is possible that several, if not all, of these factors play a role to an extent, and/or that specific subtypes of disease are due to specific underlying biological pathways; research continues to elucidate these mechanisms.

One approach to study the underlying biological pathways leading to a complex disease such as endometriosis (the development of which is determined by multiple genetic and environmental factors) is to study the effect of genetic variants on disease causation. The involvement of genetic factors in the development of endometriosis is supported by different studies [12–14]. Twin studies have shown increased concordance in monozygotic twins when compared to dizygotic twins [15, 16]; the largest such study carried out to date among Australian 3,096 female twins concluded that about 51 % of the variation in endometriosis risk is heritable [16]. In addition to human studies, familial aggregation of spontaneous (i.e., non-induced) endometriosis also has been shown in nonhuman primates such as the rhesus macaque [17].

Study designs to search for genes underlying endometriosis can be separated into hypothesis-based and hypothesis-free approaches. Hypothesis-based “candidate gene” studies typically rely on prior biological hypotheses/knowledge of the disease or of the approximate genomic location of a disease-predisposing variant derived from a hypothesis-free study. Hypothesis-free studies search the entire genome to identify disease-predisposing variants, without knowledge of their functional relevance. This review summarizes the evidence from the most recent studies investigating the genetic variation contributing to endometriosis. Of particular focus will be the results from large-scale collaborative genome-wide approaches, which have started to provide new insights into potential pathways leading to endometriosis.

## Hypothesis-Based Research: Candidate Gene Association Studies

Historically, the search of genes contributing to susceptibility of many complex diseases such as endometriosis began with hypothesis-driven “candidate gene association” studies (CGASs). Although hypothesis-free approaches have since taken off, candidate gene studies based on a biological “hunch” alone are still commonplace. CGASs are based on the a priori selection of genes with inferred biological function and association of variants in these genes with disease risk. With limited budgets and faced with over 20,000 genes in the human genome, the approach seems an attractive one. However, CGASs are inherently limited by current knowledge of biological mechanisms underlying the studied phenotype, which is attenuated in diseases where the underlying mechanisms are not very well understood, such as in endometriosis. Furthermore, to fully test the involvement of a biological pathway, one would need to investigate all genes making up the pathway (as well as factors regulating their expression), in a large sample of cases and controls; a costly approach that has, to our knowledge, never been adopted. Therefore, the probability of success of a CGAS as commonly conducted, based on the testing of a few, selected, variants in a handful of genes, is extremely low. This probability may be greatly improved if evidence of the likely genomic location of a disease-predisposing variant, derived from a whole genome linkage study for example, is included, although this approach has its own drawbacks (described later in this article).

Association studies these days typically focus on the most abundant genetic variants in the genome: single nucleotide polymorphisms (SNPs), which comprise over 90 % of all common genetic variation in the human genome [18]. SNPs are changes to the DNA at one nucleotide base pair, and can have two identities, or “alleles.” Association studies compare the frequency of the alleles of a genetic variant between cases and controls, and can be of direct and indirect design. Direct association studies aim to test specific genetic variants by genotyping these, whereas indirect association studies type a set of common (population allele frequency > 0.05) SNPs carefully selected on the basis of local genomic “linkage disequilibrium” (LD). LD is the nonrandom association of genetic variants in a population. This phenomenon means, in simple terms, that an SNP can predict the status of a genetic variant nearby because of common ancestry of that particular genomic segment. The International HapMap Project [19] has mapped LD patterns across the human genome in a number of ethnic populations, which has enabled the investigation of whole sections of the genome for their association with many common complex diseases; by testing those SNPs that are highly predictive of the status of other SNPs in the region (termed “tagSNPs”), all common genetic variation present in the population can be tested with very high coverage. Genome-wide association studies (GWASs) are of indirect design; they utilize LD by typically genotyping ~500,000–1,000,000 SNPs that tag a large proportion of the roughly 10 million common SNPs present in the human genome. Most CGASs of endometriosis have been of direct design (i.e., a limited number of specific genetic variants were genotyped and tested for association, although indirect tag approaches have also been used [20]). For a detailed review of the design of CGASs and GWASs and their underlying principles, see Zondervan & Cardon [21].

We previously published a review of CGASs published up to April 1, 2008 [20]. For this paper, we identified CGASs of endometriosis published from April 1, 2008 to April 1, 2012, conducting a systematic literature search in PubMed for English language publications, using the terms “endometriosis” with “genetics” or “genes” or “polymorphisms.” An overview of these recent CGASs is presented in Table 1. Variants from 73 candidate genes were tested (Table 1), involved in sex steroid biosynthesis and signaling pathways, adhesion molecules and matrix enzymes, immunological mechanisms, inflammatory pathways, estradiol metabolism, growth factor systems, cell cycle regulation, oncogenes, apoptosis, and angiogenic factors [22].

**Table 1:** Candidate gene association studies of endometriosis published from April 2008 to April 2012, and total evidence for these from all studies published until 2012

Candidate gene^**^	Chr	Number of studies with association 2008–2012^*^		
		Significant (*n* cases, *n* controls)	Nonsignificant (*n* cases, *n* controls)	#Sign/Total up to 2012, *n/n* ^***^
Adhesion molecules and matrix enzymes				
*CDH1*	16q22	Indian (715, 500) [73]	–	2/2
		Japanese (511, 498) [74]		
*ICAM1*	19p13	Iranian (84, 120) [75]	Korean (390, 351) [76]	2/5
*MMP2*	16q12	Estonian (150, 199) [77]	–	2/3
*MMP9*	20q11-13	Estonian (150, 199) [77]	–	1/2
*MUC2*	11p15	Taiwainese (195, 196) [78]	–	1/1
*MUC4*	3q29	Taiwainese (140, 150) [79]	–	1/1
Apoptosis, cell-cycle, DNA repair, and oncogenes				
*AKT1*	14q32	–	Indian (30, 30) [80]	0/1
*APE1*	14q11-12	–	Turkish (52, 101) [81]	0/1
*CDKN1A*	6p21	–	Taiwainese (180, 330) [82]	0/2
*CDKN1B*	12p13	Brazilian (104, 109) [83]	–	1/1
*HOGG1*	3p26	–	Turkish (52, 101) [81]	0/1
*PI3KCA*	3q26	–	Indian (30, 30) [80]	0/1
*TP53*	17p13	Mexican (151, 204) [84]	Brazilian (19, 19) [86]	4/9
		Caucasian, Asian (749, 857)^a^ [85•], Taiwainese (180, 330) [82]		
*XPD*	19q13	–	Turkish (52, 101) [81]	0/1
*XPG*	13q33	–	Turkish (52, 101) [81]	0/1
*XRCC1*	19q13	–	Turkish (52, 101) [81]	0/1
*XRCC3*	14q32	–	Turkish (52, 101) [81]	0/1
Cytokines/inflammation				
*C3*	19p13	Puerto Rican (214, 147) [87]	–	1/1
*CTLA4*	2q33	–	Brazilian (177, 172) [88]	0/2
*DR4/5*	8p21	–	Korean (138, 214) [89]	0/1
*FCRL3*	1q21-22	Brazilian (167, 167) [90]	–	0/1
*IFNG*	12q24	Korean (622, 442) [91]	–	3/3
		Indian (302, 324) [92]		
*IL10*	1q31-32	Danish (100, 358) [93]	–	5/6
		Taiwainese (196, 397) [94]		
		Chinese (214, 160) [95]		
*IL16*	15q26	Chinese (230, 203) [96]	–	1/1
*IL18*	11q22	Turkish (135, 84) [97]	–	1/2
*IL1B*	2q13-21	–	Turkish (118, 78) [98]	0/4
			Taiwainese (196, 397) [94]	
*IL2RB*	22q13	–	Korean (237, 164) [99]	1/2
*IL6*	7p21-15	–	Taiwainese (196, 397) [94]	0/5
			Korean (390, 351) [76]	
*LOXL4*	10q24	Puerto Rican (214, 147) [87]	–	1/1
*NFKB1*	4q24	Chinese (206, 365) [100]	–	1/1
*PTGS2*	1q25	–	Taiwainese (196, 397) [94]	0/1
*PTPN22*	1q13	Brazilian (140,180) [101]	Caucasian (789, 351) [103]	3/4
		Polish (171, 310) [102]		
*TCRB*	7q34	–	Italian (70, 120) [104]	0/1
*TNFA*	6p21	Indian (245, 85) [105]	Korean (105, 101) [106]	1/2
*TNFR1*	12p13	–	Korean (105, 101) [106]	0/1
*TNFR2*	1p36	–	Korean (105, 101) [106]	0/2
*TNFRSF11B*	8q24	–	Korean (138, 214) [89]	0/1
*TNFSF13B*	13q32-34	Dutch (87, 107) [107]	–	1/1
*TRAIL*	8p22-21	–	Korean (138, 214) [89]	0/1
Estradiol metabolism				
*COMT*	22q11	–	Brazilian (198, 168) [108]	0/5
			Caucasian (256, 567) [109]	
*HSD17B1*	17q11-21	Estonian (150, 199) [110]	Caucasian (256, 567) [109]	3/4
*HSD17B2*	16q24	–	Caucasian (256, 567) [109]	0/1
Growth factor systems				
*BNC2*	9p22.2	–	Caucasian (789, 351) [111]	0/1
*FGF1*	5q31	–	Chinese (421, 421) [112]	0/1
*FGF2*	4q26	Chinese (421, 421) [112]	–	1/1
*IGF1*	12q23	–	Korean (128, 108) [113]	0/1
*IGF1R*	15q26	–	Korean (128, 108) [113]	0/1
*IGF2*	11p15	Korean (128, 108) [113]	–	1/1
*TGFB1*	19q13	–	Korean (485, 352) [114]	1/4
			Korean (131, 107) [115]	
*VEGF*	6p21-12	Turkish (98, 94) [116]	Australian (958, 959)^c^ [26•]	10/12
		Chinese (344, 360) [117]		
		Caucasian (186, 180) [118]		
		Estonian (150, 199) [110]		
		Japanese, Australian, Spanish, Chinese, Estonian (1785, 1879)^b^ [25•]		
Hormone receptors				
*ESR1*	6q24-27	Chinese (214, 160) [95]	Caucasian (256, 567) [109]	7/12
		Estonian (150, 199) [110]		
*ESR2*	14q21-22	Brazilian (108, 210) [119]	Caucasian (256, 567) [109]	4/6
		Brazilian (136, 209) [120]		
		Estonian (150, 199) [110]		
*PGR*	11q22-23	Caucasian (348, 5820)^d^ [121•]	Caucasian (256, 567) [109]	6/9
Human leukocyte antigen system and immune components				
*CCL21*	9p13	Caucasian (789, 351) [103]	–	1/1
*CD40*	20q12-13	–	Caucasian (789, 351) [103]	0/1
*HLA region*	6p21	–	Japanese (89, 136) [122]	0/1
*HLA-DRB1*	6p21	Caucasian (789, 351) [103]	–	1/2
*IRF5*	7q32	–	Caucasian (789, 351) [103]	0/1
*STAT4*	2q32	–	Caucasian (789, 351) [103]	0/1
*TRAF1-C5*	9q33-34	–	Caucasian (789, 351) [103]	0/1
*VDR*	12q13	–	Brazilian (132, 195) [123]	0/1
Other enzymes and metabolic systems				
*eNOS*	7q36	Korean (299, 459) [124]	Indian (232, 210) [125]	2/4
*IRS2*	13q34	Turkish (135, 135) [126]	–	1/1
*LHB*	19q13	Brazilian (110, 209) [127]	–	1/3
*SERPINE*	7q21-22	Brazilian (140, 148) [128]	Italian (368, 329) [129]	2/3
Steroid-synthesizing enzymes, detoxifying enzymes, and receptors				
*CYP17A1*	10q24	Turkish (46, 39) [130]	Caucasian (256, 567) [109]	1/9
			Italian (104, 86) [131]	
*CYP19A1*	15q21	Caucasian (256, 567) [109]	–	4/6
		Italian (104, 86) [131]		
*CYP1A1*	15q24	–	Caucasian (256, 567) [109]	1/7
*CYP1A2*	15q24	–	Caucasian (256, 567) [109]	0/1
*CYP2C19*	10q24	Turkish (50, 50) [132]	Turkish (46, 39) [130]	1/2
*GSTM1*	1p13	Iranian (120, 200) [133]	–	7/13
*GSTP1*	11q13	–	Korean (260, 164) [134]	1/3
*PPARG*	3p25	Korean (446, 427) [135]	–	3/3

*Chr* chromosome

^*^When more than one polymorphism was investigated in a study, the result is indicated as significant if one or more of the variants were reported as significant by the authors. ^**^ Candidate gene categories are as suggested by Falconer et al. [22]. ^***^ Total number of candidate studies is summed together with data from our review in 2008 [20]

^a^Literature-based meta-analysis of *P53* codon 72 polymorphism from six published studies of Asian and Caucasian ancestry. The study showed significant association of the polymorphism with endometriosis only in the Asian patients [85•]

^b^Literature-based meta-analysis of five polymorphisms in the *VEGF* gene from 11 studies from Chinese, Indian, Korean, Japanese, Spanish, Turkish, Estonian, and Australian patients. The study identified one significant polymorphism with endometriosis from the overall meta-analysis [25•]

^c^Genotyped and tested the association of four *VEGF* polymorphisms with endometriosis in a large Australian sample. Then, performed a meta-analysis with four additional published studies and showed no evidence for association neither in the genotyped association analysis they have performed, nor in the meta-analysis for any of the four *VEGF* polymorphisms [26•]

^d^An international ovarian cancer consortium, including eight ovarian cancer case-controls studies from Australia, Europe, and the United States, investigated the association of polymorphisms in *PGR* with endometriosis in 348 endometriosis patients and 5812 control patients [121•]

For evidence of association to be credible, it is paramount that the observed associations should be replicated in an independent sample from the same underlying ethnic population [21, 23]. Generally, many reported associations do not show significant association in independent studies. This can be due to many factors, such as differences in disease definition, inadequate control selection, low coverage of the candidate genes, small sample size of the initial dataset (creating a false-positive result) or of the replication dataset (creating a false-negative result), or population differences between the discovery and the replication datasets. Moreover, to avoid failure to replicate due to Winner’s curse (the statistical phenomenon in which the effect size [the odds ratio] of the association in the discovery dataset is larger than in any subsequent replication dataset), the replication studies need to be larger in sample size and have greater power than the discovery study to detect the effect of the putative association [24].

As can be seen from the results in Table 1, no candidate gene has been robustly associated with endometriosis across studies. One of the most frequently investigated and plausible genes, encoding for vascular endothelial growth factor (*VEGF*), probably shows the most suggestive evidence of association. A literature-based meta-analysis of five polymorphisms from 11 studies including 1785 cases and 1879 control patients of Chinese, Indian, Korean, Japanese, Spanish, Turkish, Estonian, and Australian origin suggested one significantly associated polymorphism (+936T/C; TT+TC vs CC: *P* = 0.02) [25•], although no such association was found in a large study of 958 Australian cases and 959 control patients (*P* = 0.31) [26•]. Similarly, our previous review of 76 studies published until April 1, 2008 concluded that none provided clear support for any genetic variant to be robustly associated with endometriosis, and that some reported results may represent true associations, but that given the small effect sizes expected, large replication datasets or meta-analyses are required. In a large GWAS of 3,194 cases and 7,060 control patients of European ancestry, all candidate genes from the 2008 Montgomery et al. review [20] were investigated for nominal evidence of association [27•]; the only gene with a nominal *P* < 10^−3^ for SNPs in the GWAS data was the gene encoding the progesterone receptor (*PGR*) on chromosome 11, but the result for the SNP in this gene was not significant in the replication stage. This does not necessarily mean that none of these candidate genes are involved in the causation of (subtypes) of endometriosis; it also could mean that even in a large study of all types of endometriosis, power is lacking to detect their effect.

As can be seen in Table 1, the recent CGASs were conducted in ethnically diverse populations. Because different populations may have different genetic contributions to a disease, and LD patterns may vary between ethnic populations, a genetic variant identified as causal in one population may not be acting as a susceptibility variant in another. Therefore, care should be taking in interpreting and comparing findings in terms of “replication.”

## Hypothesis-Free Research: Whole Genome Linkage Studies and Their Follow-up

One of the two hypothesis-free approaches to investigate underlying genetic etiology of a disease is linkage mapping, which adopts genetic investigation of families with multiple affected individuals. This methodology became popular in the 1980s after the discovery of highly variable genetic markers (“microsatellites”), and works by 1) genotyping about 400 microsatellites across the genome; 2) comparing whether the allelic status of these variants are shared between affected people in a family; 3) summing this evidence of sharing across multiple families; 4) calculating whether the amount of sharing is more than expected by chance based on simple Mendelian laws of inheritance; and 5) identifying those genomic regions where excess sharing is statistically significant. The linkage approach was highly successful in identifying genetic variants responsible for rare, monogenic disorders. Such disorders show clear Mendelian segregation patterns through families [28, 29], and the presence of the causal genetic variant determines whether or not the disease in question will develop. The genetic causes of many of these monogenic diseases could be ‘mapped’ by investigating a handful of extended families with many affected members.

Mendelian disorders are very different from complex diseases such as endometriosis, in which one genetic variant only carries susceptibility to disease (not 100 % risk). Many more families with multiple affected women are required to conduct a linkage study of endometriosis. Linkage studies are fundamentally different from association studies; the two approaches are complementary. In contrast to association studies, which focus on SNPs common in the general population, linkage studies are designed to detect disease-causing variants responsible for disease in a family, but that are otherwise rare in the general population. Moreover, because linkage studies use recombination events in families, the resolution of the approach is very large compared to association studies: a significantly linked region typically extends across 10–50 Mb and contains hundreds of genes, in contrast to the high resolution of association studies which (depending on the local LD structure) are able to pinpoint a signal to within 10–500 Kb.

The largest genome linkage scan to date was conducted by the International Endogene Study (consisting of 1,176 affected sib-pairs from Australian and United Kingdom families), which identified a region of significant linkage on chromosome 10q26 [30]. In a subsequent analysis involving a subset of 248 families with 3 or more affected members, a second region was found on chromosome 7p13-15 likely due to one or more rare variants following near-Mendelian patterns of inheritance [31]. In a recent study, the linkage peak on chromosome 10q26 region was extensively genotyped using 11,984 SNPs in 1,144 familial cases and 1,190 control patients. The study identified three independent signals in the region, at 96.59 Mb (rs11592737), 105.63 Mb (rs1253130), and 124.25 Mb (rs2250804), with nominally significant evidence of association. However, only rs11592737 in the cytochrome P450 subfamily C (*CYP2C19*) gene replicated (*P* = 0.04) in an independent sample of 2,079 cases and 7,060 control patients [32•]. *CYP2C19* is a plausible candidate for endometriosis because it is involved in the metabolism of drugs and E including conversion of E2 to estrone (E1), and the production of E1 and E2 2a- and 16a-hydroxylation metabolites [33, 34]. Future studies should follow up by investigating novel rare genetic variants in this region and conducting gene expression analyses to further understand the role of *CYP2C19* in endometriosis.

As a first approach to follow-up the significant linkage peak on chromosome 7 region, the coding regions and upstream regulatory regions of three promising candidate genes (*INHBA*, *SFRP4*, and *HOXA10* with known roles in endometrial development) located within or close to the linkage signal were sequenced to identify potential causal polymorphisms. Sequencing, rather than genotyping, was chosen because the signal was likely due to one or more novel rare variants not present on any genotyping array. Sequencing was conducted in 47 cases from the 15 families contributing most to the linkage signal, and 11 variants were found. The minor allele frequencies (MAFs) of observed variants were compared with MAFs from two publicly available reference populations of European ancestry: 60 individuals in HapMap and 150 individuals in the 1000 Genomes Project. Five of the 11 variants were common (MAF > 0.05); the remaining six variants were rare and unlikely to be individually or cumulatively responsible for the linkage signal. These results indicate that the coding regions of these three genes do not harbor rare mutations responsible for linkage to endometriosis in these families [35]. However, this does not exclude the possibility that variants responsible for the linkage signal exist in noncoding regions of these three genes, or that they are located in other genes in this region.

## Hypothesis-Free Research: Genome-wide Association Studies

GWASs are based on the premise that common diseases such as endometriosis are caused by genetic variants that are common themselves (Common disease-common variant hypothesis [CDCV]). The first and seminal papers using GWAS methodology successfully were published in 2005–2007 [36–38], and since then the method has taken off, resulting in the detection of many common genetic variants associated with complex diseases. The NHGRI (National Human Genome Research Institute) GWA Catalog (www.genome.gov/GWAStudies) [39] counted the number of published significant genome-wide associations (*P* ≤ 5 × 10^−8^) up to June 2011 at 1,449 for 237 traits. Key developments that enabled GWASs were (1) the documentation of hundreds of thousands of common SNPs, and the LD patterns between them in different populations in the human genome by the HapMap Consortium [19]; and 2) the development of high-throughput genomics platforms capable of genotyping over 1 million SNPs in one assay at ever decreasing cost.

The first GWASs of endometriosis were published in 2010 and 2011: two in women of Japanese ancestry [40•, 41] and one in women of European ancestry [27•]. Only two [27•, 40•] reported genome-wide significant signals, which were replicated (Table 2). The first Japanese endometriosis GWAS included 1,423 cases and 1,318 control patients in the discovery sample, with cases a mixture of surgically confirmed and clinically diagnosed women. After the application of SNP quality control criteria, 460,945 SNPs were included in the discovery analysis. A replication analysis was conducted in an independent set of 484 cases and 3,974 control patients, in which the top 100 most significant SNPs from the discovery set were genotyped. A significant association for one SNP was found, rs10965235, in *CDKN2BAS* on chromosome 9p21 (*P* = 6.79 × 10^−6^, odds ratio (OR) = 1.56 [1.29–1.89], *P* = 4.89 × 10^−4^). Combining the discovery and replication samples provided a genome-wide significant result for rs10965235 (*P* = 5.57 × 10^−12^, OR = 1.44 [1.30–1.59]). Rs10965235 is located in intron 6 of *CDKN2BAS,* which encodes for the cyclin-dependent kinase inhibitor 2B antisense RNA (discussed further in this article). Uno et al. [40•] observed a second interesting potential association with rs16826658, only providing suggestive evidence, on chromosome 1p36 in a region close to *WNT4* (combined datasets: *P* = 1.66 × 10^−6^, OR = 1.20 [1.11–1.29]). *WNT4* encodes for wingless-type MMTV integration site family, member 4. Both these variants on chromosome 9p21 and 1p36 are novel susceptibility loci for endometriosis in the Japanese population [40•], and are discussed further in this article.

**Table 2:** Summary of significant genetic variants discovered by GWASs of endometriosis

Study	Novel loci	Top associated SNP	Position	Chr	Population	Sample size (Ca:Co), *n:n*	Rep	Biological explanation
Uno et al. [40•]	*CDKN2BAS*	rs10965235	Genic	9	Japanese	1,423: 1,318	Yes	Silencing of tumor suppressor gene, *CDKN2A*, by *CDKN2BAS* may have an important role in the development of endometriosis
Painter et al. [27•] and Uno et al. [40•]	*WNT4*	rs16826658	Genic	1	Japanese	1,423: 1,318	Yes	Plays a role in development of the female reproductive tract, ovarian follicle development and steroidogenesis
					Caucasian	3,194: 7,060		
Painter et al. [27•]	*mir-148a, NFE2L3, HOXA11*	rs12700667	Intergenic	7	Caucasian	3,194: 7,060	Yes	Variant associated with WHR as a measure of fat distribution. Fat distribution is also hormonally regulated; suggestive pleiotropic locus

*GWAS* genome-wide association studies; *SNP* single nucleotide polymorphism; *Ch* chromosome; *Ca* cases; *Co* controls; *Rep* replication; *WHR* Waist to hip ratio; *Ref* reference

A second, independent, Japanese GWAS was published recently, comprising a meta-analysis of two GWAS on two case–control datasets. After quality control, 282,828 SNPs were tested for association in 696 endometriosis cases (not all surgically confirmed) and 825 control patients. Limited by their sample size, their aim was to detect potential common susceptibility loci with large effect on the disease. The study did not reveal any significant susceptibility loci for endometriosis, which is likely to be due to the small sample size of their dataset [41].

The largest GWAS on endometriosis to date was performed in women of European ancestry by the International EndoGene Consortium (IEC) [27•]. The study involved 3,194 surgically confirmed endometriosis cases and 7,060 control patients from Australia and the United Kingdom. Disease severity was assessed retrospectively from surgical records using the rAFS classification system and grouped into two phenotypes: stage A (stage I or II disease or some ovarian disease with a few adhesions; *n* = 1,686, 52.7 %) or stage B (stage III or IV disease; *n* = 1,364, 42.7 %), or unknown (*n* = 144, 4.6 %). After quality control, analyses were performed using 504,723 SNPs. Analyzing all SNPs combined, Painter et al. [27•] showed a significantly increased genetic loading among 1,364 cases with stage B endometriosis compared to 1,666 with stage A disease (proportion of endometriosis variation explained by common SNPs: 0.34 [SD: 0.04] vs 0.15 [SD: 0.15] respectively; *P* = 1.8 × 10^−3^). Because of this result, two GWA analyses were performed, using (1) 3,194 “all” endometriosis cases and (2) 1,364 stage B cases. For “all” endometriosis, the strongest signal observed was rs12700667 in an intergenic region on chromosome 7p15.2 (*P* = 2.6 × 10^−7^, OR = 1.22 [1.13–1.32]), which was considerably stronger when limiting cases to those with stage B endometriosis (*P* = 1.5 × 10^−9^, OR = 1.38 [1.24–1.53]). A second strong association was found for rs1250248 (2q35) within *FN1* (*P* = 3.2 × 10^−8^). In the replication phase, 70 SNPs that produced nominal evidence of association with “all” or stage B were genotyped in an independent dataset comprising 2,392 self-reported surgically confirmed cases and 2,271 control patients from the Nurses’ Health Study I and II in the USA. The association on 7p15.2 with rs12700667 was replicated (*P* = 1.2 × 10^−3^, OR = 1.17 [1.06–1.28]). However, there was no evidence for replication of rs12540248 (*FN1*) or association with the remaining SNPs. Combined analysis of all 5,586 cases and 9,331 control patients from Australian, UK and US datasets further confirmed association between “all” endometriosis and 7p15.2 (rs12700667, *P* = 1.4 × 10^−9^, OR = 1.20 [1.13–1.27]). Of note is that the estimated percentage of “all” endometriosis variance explained by rs12700667 was only 0.69 % of the estimated 51 % heritability. Rs12700667 is located in a roughly 924-kb intergenic region containing at least one noncoding RNA (AK057379), predicted transcripts and regulatory elements, and a micro RNA (miRNA [hsa-mir-148a]) about 88 kb upstream (discussed further in this article).

Painter et al. [27•] also investigated the associations reported by Uno et al. [40•] in Japanese women. They found no evidence for association with rs10965235 on chromosome 9p21 (rs10965235 is monomorphic in individuals of European descent, reflecting the different genetic (ancestral) backgrounds between the studies), nor with any SNPs in LD with rs10965235. However, there was evidence for replication of rs7521902 on 1p36, close to *WNT4* gene, with the strongest signal for stage B endometriosis (*P* = 7.5 × 10^−6^, OR = 1.25, 95 % CI 1.13–1.38); meta-analysis of the evidence from “any” endometriosis (as severity of disease was not assessed in the Japanese GWAS) combining the three GWAS datasets resulted in a genome-wide significant *P* value of 4.2 × 10^−8^ (OR = 1.19, 95 % CI 1.12–1.27).

GWASs for endometriosis, to date, have been conducted on samples of relatively modest sizes compared to other conditions, but these first studies have shown that there are no common variants with large effects. ORs for replicated, genome-wide significant signals are all lower than 1.5, an observation that mirrors results from the many GWAS performed on complex diseases to date. The results from Painter et al. [27•] suggest that future larger studies enriched for surgically confirmed rAFS stage III/IV cases will be better powered to identify risk loci of endometriosis, but further work, requiring larger samples, needs to be conducted to assess whether specific severe subtypes such as deep infiltrating endometriosis [42] or rectovaginal disease [43] are genetically heterogeneous from each other, or indeed if rAFS stage III/IV is genetically heterogenous with respect to presence of endometriomas or scarring/adhesions. Furthermore, the implicated variants from GWASs are likely to be in LD with the actual disease-causing variants, and further in-depth work to explore genetic variation in the regions, as well as their influence on gene expression and downstream biological pathways, now needs to be conducted. The variants themselves are of small effect and explain only a very small proportion of disease prevalence; hence, they are unsuitable individually to serve as diagnostic markers. However, they provide important data to define the underlying pathways contributing to the disease, can be used to define genetically heterogeneous subtypes that are likely to respond differently to treatments, and thus, through the identification of differential pathways, help in the development of diagnostic tests and future treatments.

### Genes Implicated by GWAS of Endometriosis to Date

Although there was no evidence of a signal in women of European ancestry [27], the GWAS by Uno et al. [40•] clearly implicated *CDKN2BAS* as potentially involved in endometriosis in Japanese women (Table 2). *CDKN2BAS* is an interesting candidate because it is expressed in the uterus and regulates the expression of *CDKN2B* (*p15*), *CDKN2A* (*p16*), and *ARF* (*p14*), which are tumor suppressor genes [44–46]. *CDKN2A* is a cell-cycle–dependent kinase inhibitor and acts as a negative cell-cycle regulator [47, 48]. Inactivation of *CDKN2A* has been reported in a subset of endometrial carcinomas [49–52]. Moreover, hypermethylation of the *CDKN2A* promoter region has been observed in endometriosis [52], and loss of heterozygosity on the *CDKN2A* locus has been found in endometriosis, suggesting that *CDKN2A* might play a role in the regulation of endometrial cell growth [53]. The evidence from the literature suggests that silencing of tumor suppressor genes such as *CDKN2A* by *CDKN2BAS* might have an important role in the development of endometriosis [53].

The second novel genetic variant, which was supported by evidence from GWASs in both women of Japanese and European ancestry, is located near *WNT4*, with evidence from the latter mainly limited to rAFS stage III/IV disease. *WNT4* is known to play an important role in the development of the female genital tract from the Müllerian duct that develops into the fallopian tubes and uterus; the loss of *WNT4* in knockout mice was shown to lead to complete absence of the Müllerian duct [54, 55]. Moreover, *WNT4* is expressed in normal peritoneum, suggesting that endometriosis may arise through metaplasia using developmental pathways involved in the development of the female genital tract [54]. Furthermore, it is possible that genetic variants in *WNT4* might contribute to endometriosis susceptibility through abnormal cell growth in female genital tract. With all the evidence from the literature, *WNT4*, through its prominent role in development of the female reproductive tract [55], ovarian follicle development, and steroidogenesis [56], might play a critical role in development of endometriosis.

The third novel genetic variant found only in the GWASs of women of European ancestry [27•], rs12700667, is located in an intergenic region on chromosome 7p15.2. This locus was not reported among the top 100 signals in the Japanese GWAS by Uno et al. [40•]; however, given their sample size they would have had only 13 % power to detect it [27•]. Chromosome 7p15.2 contains multiple genomic features that may be involved in endometriosis development. The region contains a number of expressed sequence tags (ESTs) and a miRNA (*has-mir-148a*), suggesting a role in gene expression regulation. Further downstream, there are several genes including *NFE2L3* (highly expressed in placenta) and two endometriosis candidate genes, *HOXA10* and *HOXA11,* which are transcription factors that play a role in uterine development. Another interesting aspect about this variant comes from a large GWAS (*n* = 77,167) investigating the genetic variants associated with fat distribution as measured by waist-to-hip ratio adjusted for the effect of body mass index (WHRadjBMI) [57]. Interestingly, rs12700667 is positioned at the same locus as one of 13 genome-wide significant loci they reported as associated with reduced WHRadjBMI, a result that is currently further being explored. As endometriosis and fat distribution are both hormonally dependent, pleiotropic genetic loci acting on both traits may well exist.

## Challenges and Future Directions

The first GWASs of endometriosis have identified three genetic loci highly likely to be involved in the pathogenesis of endometriosis in women of Japanese and European ancestry. Although they have identified the loci, they have not pinpointed the actual genetic variants that are causal to endometriosis. Furthermore, they explain only a fraction of the heritability of endometriosis. Therefore, directions for further investigation include the following:

1. The GWASs of endometriosis to date are relatively crude, in that they generally only describe associations with endometriosis, without regard for subtypes or differences in symptomatology (pain and infertility). Only the IEC GWAS [27•] considered stage B versus stage A endometriosis, showing that the two are genetically different in etiology. Future studies need to include cases who are phenotyped in much greater detail to enable distinction between endometriosis subtypes. For these studies to have sufficient power, however, even larger sample sizes are required than used to date.
2. Of the 1,449 novel loci identified by GWASs up to June 2011 [39], the number of loci identified per complex trait varies greatly. Visscher et al. [58] showed that the number of discovered variants is strongly correlated with the sample size of different studies. The sample sizes of the endometriosis GWASs are at the lower end of those that have been successful in identifying loci involved in the etiology of a complex trait. This means that increasing the discovery sample size will increase the number of discovered variants, which also was suggested by prediction analyses based on all genotyped SNPs in the GWAS of the IEC [27•]. When individual causal variants only explain a small amount of variation, then the power to detect them is low in studies with small sample sizes. Therefore, there is a need for larger endometriosis GWASs in different populations, and meta-analyses of GWASs, to detect additional common causal variants with modest effect on risk. As mentioned before, the power of these studies can be increased by focusing on more severe subtypes, and in general by ensuring detailed and accurate clinical phenotyping allowing the investigation of such subtypes.
3. To maximize information gained from GWASs, dense genotyping platforms containing at least 500,000 SNPs selected on the basis of LD should be utilized in GWASs, and imputation approaches should be used to estimate non-genotyped common variants (eg, using 1000 Genomes project data [http://www.1000genomes.org]). To follow up on initial GWA scans and to further improve the coverage, custom-made gene arrays could be designed including dense SNP sets focused on implicated genes (although, if budget allows, sequencing of these may be preferable); these could be supplemented by genes robustly implicated in hypothesized related common disease such as gynecological cancers [59, 60], migraine [61] or complex traits such as age of menarche [62] and body mass index (BMI) [63]. The use of whole exome genotyping arrays, including carefully selected sets of common and low-frequency variants in exons, also could be a promising approach to identify additional signals.
4. Although GWASs are unbiased by prior biological knowledge or genomic location, they are limited with regard to their ability to only assess the effect of common SNPs (MAF > ~0.05) in the general population. It is possible that some of the unexplained genetic variation may be due to rarer variants (MAF < 0.5 %), either single-site or structural, that are not captured by current GWA genotyping arrays [64, 65]. Indeed, the effects of structural variation are generally missed out in GWAS (although some of the GWA arrays contain selected probes to test for common copy number variation [CNV]). Rare and structural variants can be investigated through exome or whole-genome sequencing; however, these studies are still challenging because of their cost, with bioinformatic methodology to accurately call structural variants in particular still under development.
5. Preliminary studies have suggested the role of gene–gene and gene–environment interactions in the development of endometriosis [66, 67]. There is no doubt that such interactions play a role in the development of many complex traits, but to robustly ascertain them without strong prior hypotheses, even larger sample sizes are generally required than advocated for GWAS. A biological route through which environmental factors could impact on the influence of genes on disease is through epigenetic modification of DNA (mainly methylation). There is considerable interest in epigenetic variants as contributors to the unexplained genetic variation of endometriosis [68]. One method through which the role of epigenetic variants in the development of endometriosis could be tested is by using a twin cohort where age-specific endometriosis concordance rates are obtained from monozygotic and dizygotic twins. If the monozygotic twin concordance rates for endometriosis increase with age, this could provide evidence for the potential role of epigenetic factors [69]. However, a major issue for endometriosis could be age-specific confounding by patterns of diagnosis. Platforms to detect genome-wide DNA methylation are being developed, and although currently still based on selected loci, are increasingly providing exciting new avenues for exploring the effect of epigenetic changes on complex disease such as endometriosis.
6. Although the study of the genetics of endometriosis is starting to reap fruits, it is still faced with many challenges inherent to the complexities of the disease. This includes the general lack of correlation between disease stage and pain severity; much greater phenotypic detail of cases included in genetic studies is needed to start distinguishing between potential subtypes of disease and symptomatologies. A particular problem is the need for a surgical diagnosis, which hampers population-based research. A complementary avenue may be to study the disease in animal models, in particular nonhuman primates such as the rhesus macaque [70] or the baboon [71, 72], with the rhesus macaque a promising model for the study of heritable, spontaneously occurring endometriosis [17]. Work on the genetics of endometriosis in the rhesus macaque, comparing human and rhesus macaque genomes, is currently underway.

## Conclusions

There is mounting evidence for genetic variants contributing to endometriosis susceptibility. To date, candidate gene studies have not provided robust, replicated genetic variants associated with endometriosis and they have low a-priori chance of success when based solely on biological hypotheses. Linkage studies in pedigrees have found some regions of interest, which are likely to harbor variants implicated in familial endometriosis; however, due to the methodology, the regions identified are large and contain many genes of potential interest. These regions require further investigations to elucidate the susceptibility variants. The much-anticipated initial GWAS results identified three regions showing robust association with endometriosis in women of Japanese and European ancestry. Although they are by far the largest studies of endometriosis to date, they were of relatively small sample size compared to GWASs of other traits, and generally lacked detailed clinical information that would have allowed investigation of more etiologically homogeneous subphenotypes of endometriosis. Such information needs to be collected systematically for future analyses, and well-designed and sufficiently powered GWASs of thousands of cases and controls need to be conducted to identify novel genetic variants associated with endometriosis. These larger studies also will allow for better investigation of potential gene–gene and gene–environment interactions. Moreover, there are upcoming opportunities to study the impact of environment on genetic factors influencing endometriosis, through epigenetic studies, with the development of platforms to detect epigenetic changes genome-wide. Identification of robust, replicated genetic/epigenetic variants of endometriosis will pave the way to functional studies to better understand the underlying mechanisms of endometriosis, leading to better diagnosis and treatment of the disease. Complimentary research in animal models, particularly nonhuman primate models such as the rhesus macaque and the baboon, which can develop endometriosis spontaneously, should help to further elucidate the genetics of this complex condition.
